# ZEB1 expression in Th17 cells correlated with p-STAT3 in human apical periodontitis

**DOI:** 10.1186/s12903-025-05633-y

**Published:** 2025-02-27

**Authors:** Xiaoyue Sun, Jingwen Yang, Zijun Wang, Qing Nie, Qian Yang, Wei Zhang, Mingwen Liu, Li Wang, Lingxin Zhu

**Affiliations:** State Key Laboratory of Oral & Maxillofacial Reconstruction and Regeneration, Key Laboratory of Oral Biomedicine Ministry of Education, Hubei Key Laboratory of Stomatology, School & Hospital of Stomatology, Wuhan University, Wuhan, 430079 China

**Keywords:** ZEB1, IL-17, Th17 cells, p-STAT3, Human apical periodontitis

## Abstract

**Background:**

ZEB1, a zinc-finger E homeobox-binding transcription factor most frequently associated with developmental programs linked to epithelial-mesenchymal transition, has been demonstrated to regulate immune cell function. The study aimed to investigate the expression pattern of ZEB1 in Th17 cells and its colocalization with p-STAT3 in human apical periodontitis lesions.

**Methods:**

Thirty-nine human periapical tissues were collected for ex vivo study, including periapical granulomas (PGs, *n* = 14), radicular cysts (RCs, *n* = 12), and healthy control tissues (control group, *n* = 13). Inflammatory infiltration of the lesions was assessed using hematoxylin-eosin staining. The expression of ZEB1 was detected and analyzed by immunohistochemistry. The localization of ZEB1 in Th17 cells and its colocalization with p-STAT3 were assessed using fluorescence colocalization.

**Results:**

ZEB1 expression was significantly higher in PGs and RCs than in the healthy control group; however no significant difference between the two groups was observed. Immunofluorescence analysis revealed that ZEB1 expression was correlated with IL17 and CD4 double-positive cells in human periapical lesions. ZEB1/ p-STAT3 double-positive cells were predominant in RCs and PGs than in the healthy control group.

**Conclusions:**

The expression of ZEB1 was significantly elevated in PGs and RCs, and correlated with Th17 cells and p-STAT3 expression. This study revealed that ZEB1 is a potential player correlated with STAT3 activation and Th17 cells in apical periodontitis pathogenesis.

**Supplementary Information:**

The online version contains supplementary material available at 10.1186/s12903-025-05633-y.

## Introduction

Periapical disease is a common inflammatory disease that originates from periradicular tissues. Periapical granuloma and periapical cyst are different stages of chronic periapical disease [1]. Clinically, root canal treatment is the primary approach for preserving the affected tooth and managing periapical disease. However, in cases where root canal treatment or retreatment fails to resolve the periapical infection, surgical endodontic intervention is often required [2]. The healing of periapical tissues and the long-term survival of the tooth are closely linked to the overall oral health of the patient. Despite advances in treatment, effective non-invasive therapy to reduce inflammation in periapical lesions remain elusive [3]. Therefore, investigating the pathogenesis of human apical periodontitis may provide valuable insights for the development of targeted therapeutic strategies.

The role of immune cells in the development of periapical inflammation and bone resorption has been widely interpreted [3]. For instance, the expression of IRF5 and IRF8 were elevated in macrophages within periapical lesions, while the expression of PINK1/Parkin, a key signaling molecule that regulates the classical pathway of mitochondrial autophagy, was significantly up-regulated [4–6]. T helper 17 (Th17) cells are a distinct subpopulation of CD4^+^ T helper cells known for their secretion of the pro-inflammatory cytokine interleukin (IL)-17 [6]. IL-17^+^ Th17 cells have been demonstrated to be present in periapical lesion tissues in both animal models and human cases [7]. Th17 cells contribute to local immune responses through the secretion of cytokines such as IL-17, IL-21, and IL-22, which promote the infiltration of inflammatory cells, including macrophages and neutrophils, thereby amplifying the inflammatory response [8]. Furthermore, IL-17 induces osteoclastogenesis, resulting in enhanced bone resorption within periapical tissues, which subsequently compromises tooth stability [9]. In addition, the functional imbalance between Th17 cells and T regulatory (Treg) cells within periapical lesions may further exacerbate the pathological progression [10].

Zinc-finger E homeobox-binding 1 is a member of the ZEBs family of transcription factors that is best known for its role in driving epithelial to mesenchymal transition (EMT) [11]. In recent years, our understanding of EMT transcription factors has broadened, and many non-EMT regulatory functions of ZEB1 have been discovered. ZEB1 is expressed by various myeloid and lymphoid immune cells, and regulates cell fate [12]. ZEB1 is a key upstream regulator of metabolic reprogramming during osteoclast activation, which controls the energy metabolism of osteoclasts and affects their bone resorptive function of osteoclasts [13]. ZEB1 regulates tumor-associated macrophage migration in hypoxic cancer and is a key factor in maintaining tumor-associated macrophages and promote caner progression [14]. ZEB1 is also required for the survival and maintenance of antiviral memory CD8^+^ T cells and promotes the differentiation of CD4^+^ helper T cell subsets Th1 and Th17 but inhibits the production of Th2 cytokines [15, 16]. A dynamic gene regulatory network evolution analysis showed that ZEB1 plays an important role in the whole process of Th17 cell differentiation [17]. However, the presence of ZEB1 in human periapical inflammatory diseases and its correlation with Th17 cells in periapical inflammatory tissues remain unknown.

Signal transducer and activator of transcription 3 (STAT3), a cytokine-responsive transcription factor, is involved in different inflammatory processes and in the development of osteodestructive diseases [18]. STAT3 activation is usually reflected in the phosphorylation of STAT3 at Tyr705, followed by the incorporation of a STAT3 dimer into the nucleus, which affects the expression level of the target gene [18]. STAT3 was overactivated in periapical inflammatory macrophages and that inhibiting STAT3 activation effectively protects mice from infection-induced periapical inflammation [19]. Activation of STAT3 signaling plays a pivotal role in inducing ZEB1-mediated epithelial-mesenchymal transition (EMT) in various tumors, with STAT3 exerting a strong influence on the expression of ZEB1 in tumor cells [20]. In colorectal cancer, STAT3 can directly bind to the promoter region of the ZEB1 gene, thereby enhancing its transcriptional activity [21]. Recent studies have provided further insights into the potential interplay between STAT3 and ZEB1 in immune- and inflammation-related pathologies [15–16, 22]. ZEB1 promotes p-STAT3 during Th17 differentiation by repressing expression of a JAK2-targeting miRNA [16]. However, whether ZEB1 and STAT3 are simultaneously expressed in the periapical inflammatory tissue cells remains unknown.

In summary, we hypothesized that the upregulation of ZEB1 expression in human periapical inflammation promotes Th17 cell activation, and ZEB1 transcription is closely associated with the activation of p-STAT3. Therefore, the aim of this study was to investigate the expression patterns of ZEB1 and its potential interaction with p-STAT3 in Th17 cells within human periapical lesions. Exploring this molecular mechanism may offer new insights into the development of non-surgical therapeutic strategies for human apical periodontitis.

## Methods

### Clinical inclusion criteria

The patients enrolled in this study were fully informed of the possible discomfort and risks and signed an informed consent form. The patients’ ages ranged from 20 to 60 years (22 males and 17 females), none of whom had any systemic diseases or had received antibiotic therapy within the last six months, and their ASA scores were classified as ASA I. Endodontic microsurgery was performed according to clinical indications for the affected tooth including incisors and premolars, at the Department of Oral and Maxillofacial Surgery. Clinical indications include: (1) failure of root canal treatment or retreatment in teeth with periapical disease, resulting in pain or swelling; (2) teeth with periapical disease that have undergone root canal treatment and are restored with a bridge abutment or cast post, that make root canal retreatment difficult; (3) symptomatic overfilling with gutta-percha or the presence of foreign material; (4) periapical lesions with a radiolucent diameter greater than 8 to 10 millimeters. Endodontic microsurgery is not performed in cases of acute or chronic exacerbation of periapical abscesses [23].

### Tissue sample collection

The diagnosis of periapical disease was determined by a combination of clinical history, radiographic appearance, and histopathology, according to the World Health Organization criteria. The periapical tissues were collected and classified as periapical granulomas (PGs) or radicular cysts (RCs). PGs is primarily composed of inflammatory cells such as macrophages, lymphocytes, and plasma cells, along with fibrous tissue [24]. In the clinical X-ray image of PGs, there is typically a radiolucent area with blurred margins, and the lesion is relatively localized [25]. RCs has a well-defined cavity and is a cystic lesion caused by the proliferation of epithelial rests in the periapical region [24]. In the clinical X-ray image of RCs, there is a distinct round or oval radiolucent area with well-defined borders, sometimes accompanied by bone expansion [25].

A total of 14 samples of PGs and 12 samples of RCs were collected during surgery. The size of all samples was recorded using a millimeter scale, with the diameter of the periapical lesion samples not smaller than 3 mm. Periapical lesions were classified according to their diameter as follows: small (3–4 mm), medium (5–6 mm), and large (> 6 mm). Gingival tissues from the extracted healthy third molars were selected as healthy controls, and 13 healthy tissues were collected. Samples were fixed in 4% buffered paraformaldehyde for 24 h, immediately after their collection. Each tissue was evenly and randomly divided into two parts using a sharp surgical scalpel, and one part was embedded in paraffin, while the other was embedded in Tissue-Tek O. C. T compound (Sakura Finetek USA, Inc., USA).

### Histologic examination

The paraffin blocks were cut into histologic sections, which were approximately 5 μm in thickness. After the sections were deparaffinized and rehydrated, hematoxylin-eosin staining was performed to distinguish between their histological types and inflammation. PGs were defined as those consisting of granulomatous tissue and infiltrating inflammatory cells located in the periapical region without stratified layered squamous epithelial cells [24]. RCs were defined as lesions located in the periapical region of dead pulp teeth with liquid or semi-solid content surrounded by stratified non-keratinized squamous epithelium [24].

The degree of inflammatory infiltration was analyzed under a 400X microscope (Olympus, Tokyo, Japan) by selecting three consecutive microscopic fields, starting from the center of inflammation of the sample to the connective tissue [4, 5]. The degree of inflammation was graded as follows: Mild infiltrate, inflammatory cells present only in the first microscopic area; Moderate infiltrate, inflammatory cells expanded into the second microscopic area; and grade Severe infiltrate, inflammatory cells extensively infiltrating all three microscopic areas [5, 6].

### Immunohistochemical staining

The following experimental steps were followed for all paraffin sections; dewaxing in xylene, passing through a gradient ethanol solution into water, followed by incubation with gastrin antigen repair solution for 30 min. Immunohistochemical staining for ZEB1 was performed using the UltraSensitive SP (Rabbit) IHC Kit (Maixin, Fuzhou, China) according to the manufacturer’s instructions. Briefly, the sections were incubation with 3% hydrogen peroxide at 37 °C for 20 min to block the endogenous peroxidase activity, and then were incubated with goat non-immune serum for 30 min at 37 °C to block non-specific antibody binding. Afterwards sections were incubated with Anti-ZEB1 antibody produced in rabbit (1:500; cat. no. SAB5701068; Sigma-Aldrich) overnight at 4 °C. After washing with phosphate buffered saline (PBS), sections were treated with biotin-labeled sheep anti-rabbit IgG polymer at 37 °C for 30 min and then incubated with streptomyces anti-biotin protein-peroxidase for 20 min at 37 °C. Subsequently, the sections were stained using the DAB Kit (cat. no. PV-9000; ZSGB-BIO, Beijing, China) for 1 min, followed by counterstaining with hematoxylin. Non-immune serum was used as a negative control. After blocking, the sections were observed under a light microscope (Olympus, Tokyo, Japan). Immuno-positive cells were quantified using ImageJ and IHC Profiler [26]. The “IHC Profiler” program is integrated into the plugin menu of ImageJ software, and the operation method follows the developer’s recommendations. After opening the image in ImageJ, the newly optimized color deconvolution plugin is utilized for the deconvolution process. Upon selecting the “H DAB” vector in the color deconvolution pop-up window, the IHC Profiler automatically generated a histogram of the DAB-stained image and displays the corresponding scoring results on the screen [26].

### Immunofluorescence labelling

The cryostat sections were air-dried and then washed with PBS three times to remove the O. C. T compound. The sections were permeabilized with 0.1% Triton X-100 for 15 min. After washing, the sections were blocked with 2.5% bovine serum albumin (BSA) at 37 °C for 30 min and incubated overnight at 4 °C with primary antibodies as follows: rabbit polyclonal antibodies against ZEB1 (1:500; cat. no. SAB5701068; Sigma-Aldrich), rat monoclonal CD4 antibody (1:200; cat. no. sc-13573; Santa Cruz Biotechnology), mouse monoclonal IL-17 antibody (1:200; cat. no. sc-374218; Santa Cruz Biotechnology) and mouse monoclonal p-STAT3 antibody (1:200; cat. no. sc-8059; Santa Cruz Biotechnology). Negative controls were incubated with non-immune bovine serum as the primary antibody. After thoroughly washed, sections were incubated with secondary antibodies as follows: Dylight 488-conjugated goat anti-mouse IgG (Abbkine), Dylight 594-conjugated goat anti-rabbit IgG (Abbkine), and Dylight 649-conjugated goat anti-rat IgG (Abbkine), at 37 °C for 1 h while avoiding light. Subsequently, the sections were infiltrated with mounting medium containing DAPI (nuclear dye, Zhongshan, China) and observed under a fluorescence microscope (Leica, Germany) or a confocal microscope (Olympus, Japan). Each sample was randomly divided into five regions and analyzed at 400X magnification (0.0714 mm^2^/field). Positive cells were counted in each area, and the density of positive cells was expressed as the number of cells per square millimeter.

### Statistical analysis

Data are presented as mean ± standard deviation (SD). Box plots were used to show the median, interquartile range, and minimum and maximum values. Significant differences between two groups were analyzed using an unpaired two-sided Student’s t-test, while one-way analysis of variance (ANOVA) was used to evaluate differences among multiple groups using GraphPad Prism 7.0 (GraphPad Software Inc.). *P*-value < 0.05 was considered significantly different.

## Results

### Immunohistochemical localization of ZEB1 in human periapical tissues

**Fig. 1:**
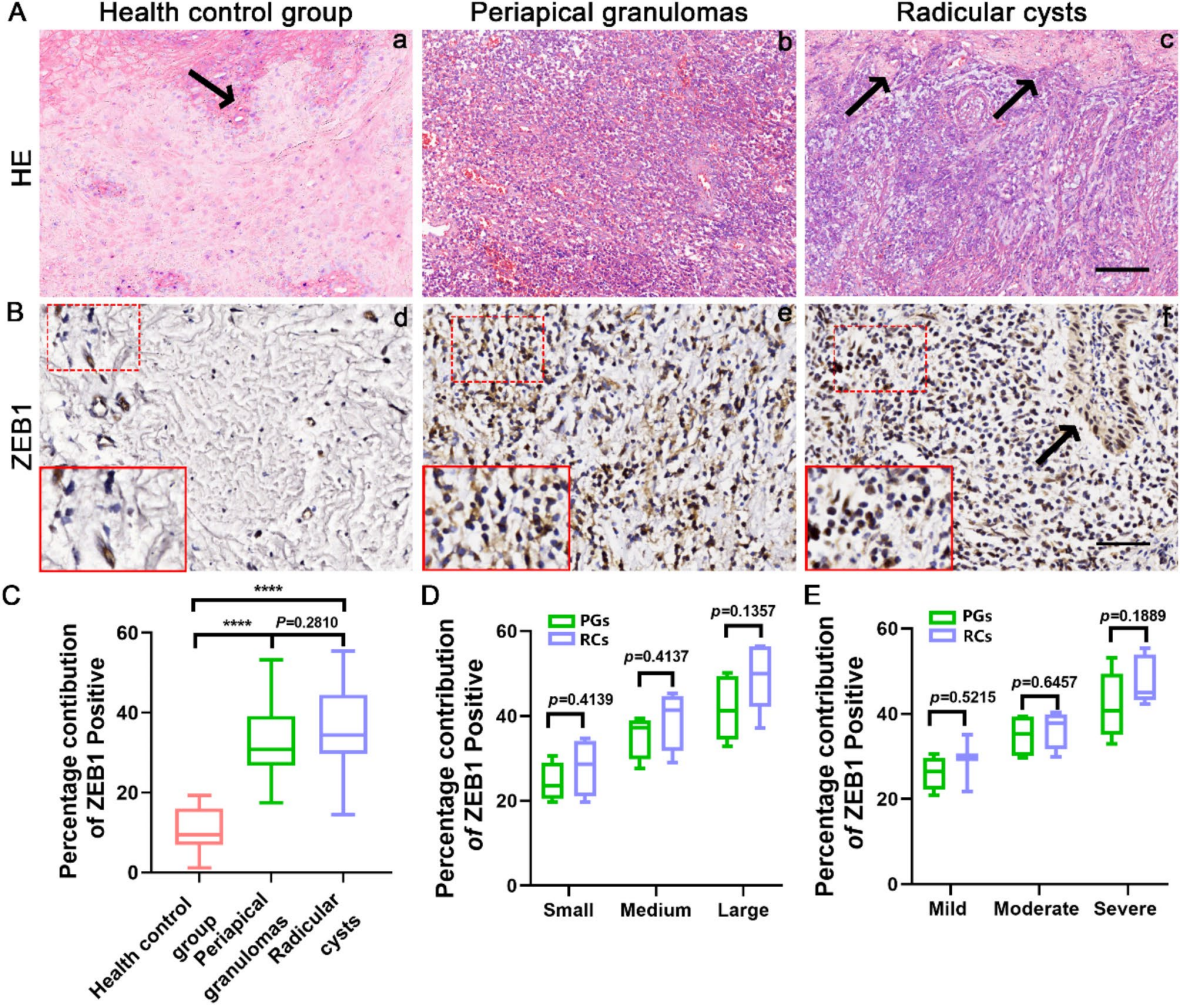
Hematoxylin-eosin (H&E) staining and immunohistochemical evaluation of ZEB1 in human healthy gingiva and apical periodontitis tissue. **(A)** Histologic analysis showing inflammatory infiltrates in (a) healthy control group, (b) periapical granulomas (PGs), and (c) radicular cysts (RCs). Scale bars = 100 μm. The black arrow indicated the area of epithelium. **(B)** Immunohistochemical staining of ZEB1 in (d) healthy control tissues, (e) PGs, and (f) RCs. Scale bars = 50 μm. **(C)** Statistical analysis of ZEB1 expression in every group via a box plot. Results are expressed as the number of *percentage contribution of* ZEB1 *positive* using IHC Profiler. ****p <.01. The *p*-value statistically significant when *p* <.05. **(D)** Statistical analysis of ZEB1 expression in different size of PGs and RCs via a box plot. Results are expressed as the number of *percentage contribution of* ZEB1 *positive* using IHC Profiler. The *p*-value statistically significant when *p* <.05. **(E)** Statistical analysis of ZEB1 expression in different inflammatory infiltration degree of PGs and RCs via a box plot. Results are expressed as the number of *percentage contribution of* ZEB1 *positive* using IHC Profiler. The *p*-value statistically significant when *p* <.05

Histological examination showed inflammatory cells in PGs and RCs, whereas healthy controls contained a large amount of fibrous tissue and very few inflammatory cells (Fig. 1A). Analysis of the degree of inflammatory infiltration in the PGs revealed six samples of severe infiltrate, four samples of moderate infiltrate, and four samples of mild infiltrate. In RCs, the inflammatory infiltrate was severe infiltrate in five, moderate infiltrate in four, and mild infiltrate in three samples. Immunohistochemical staining showed a higher expression of ZEB1 in PGs and RCs than in healthy controls (Fig. 1B). ZEB1-positive cells were dark brown in color, with strong staining in the nuclei and cytoplasm. The expression of ZEB1 was mainly in the vascular endothelial cells of healthy gingival tissue. However, in the periapical lesions, ZEB1 expression was closely associated with inflammatory cells, exhibiting both focal and dispersed patterns. In PGs, ZEB1-positive cells were primarily inflammatory cells, including lymphocytes and macrophages. Notably, in RCs, ZEB1 was expressed not only in inflammatory cells but also in epithelial cells. Statistical analysis of *Percentage contribution of positive* for each sample using Image J and IHC Profiler revealed that *Percentage contribution of positive* was significantly higher in the PGs and RCs groups than in healthy controls, while there was no significant difference between PGs and RCs (Fig. 1C).

To further explore the expression pattern of ZEB1 in periapical tissues, the expression of ZEB1 in PG and RC with different lesion sizes was statistically analyzed (Fig. 1D). The expression level of ZEB1 in both PG and RC increased with the size of the lesion, with the highest expression observed in large lesions. In RCs of the same size, the ZEB1 expression level was slightly higher than in PG, likely due to the expression of ZEB1 in epithelial cells within RCs. Furthermore, the expression of ZEB1 in PGs and RCs at different levels of inflammatory infiltration were analyzed (Fig. 1E). ZEB1-positive cells were fewer in PGs and RCs with mild infiltrate compared to those with moderate infiltrate. Additionally, PGs and RCs characterized by severe infiltrate exhibit the highest levels of ZEB1 expression. Notably, RCs with severe infiltrate showed a slightly higher number of ZEB1-positive cells than PGs with the same degree of infiltration. However, no significant differences in ZEB1 expression were observed between PGs and RCs at different levels of inflammatory infiltration.

### Colocalization of ZEB1 with IL-17 and CD4 in infiltrated Th17 cells of human periapical tissues

**Fig. 2:**
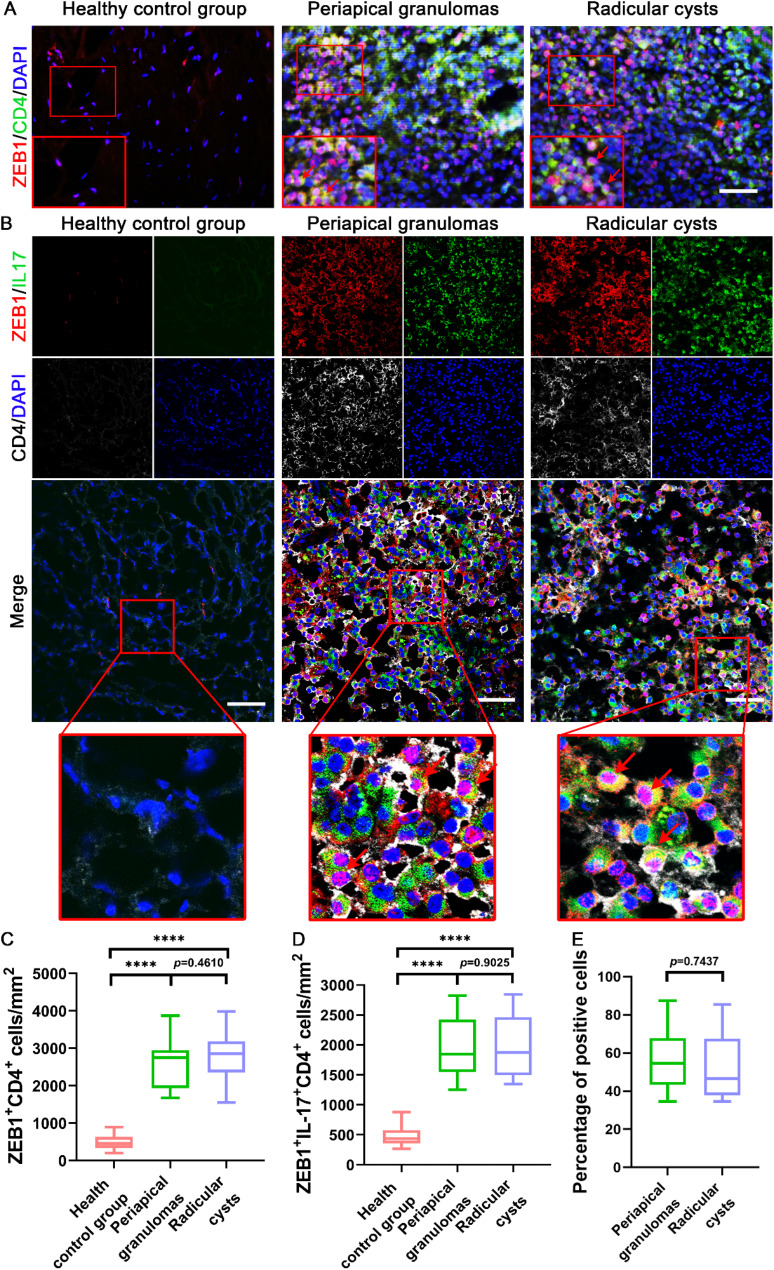
Immunofluorescence colocalization of ZEB1 with IL-17 and CD4 in human periapical tissues. **(A)** ZEB1-CD4 double labeling in healthy control group, PGs, and RCs. *Red*, ZEB1; *green*, IL17; *White*, CD4; *blue*, DAPI. Scale bars = 50 μm. **(B)** ZEB1-IL17/CD4 triple labeling in (a) healthy control group, PGs, and RCs. *Red*, ZEB1; *green*, IL17; *White*, CD4; *blue*, DAPI. Scale bars = 50 μm. **(C)** Statistical analysis of ZEB1-CD4 double-positive cells in every group via a box plot. Results are expressed as the number of positive cells per square millimeter. ****p <.0001. The *p*-value statistically significant when *p* <.05. **(D)** Statistical analysis of ZEB1-IL17/CD4 triple positive cells in every group via a box plot. Results are expressed as the number of positive cells per square millimeter. ****p <.0001. The *p*-value is statistically significant when *p* <.05. **(E)** Statistical analysis of ZEB1-positive cells among IL17^+^CD4^+^ Th17 cells in every group via a box plot. Results are expressed as the percentage of positive cells. ****p <.0001. The *p*-value is statistically significant when *p* <.05

T-helper 17 (Th17) cells are a subset of CD4^+^ helper T cell [7]. We first detected the colocalization of ZEB1 with CD4 in human periapical lesions (Fig. 2A). IL-17 is a hallmark of the inflammatory cytokine produced by Th17 cells [7]. We further explored the expression of ZEB1 in Th17 cells in human periapical tissues (Fig. 2B). Many ZEB1-CD4 double-positive cells infiltrated the PGs and RCs, whereas in healthy gingival tissue, there were little ZEB1-CD4 positive cells (Fig. 2C). Almost no IL-17 or CD4-positive cells were observed in the healthy tissues. However, large infiltration of IL17 and CD4 double-positive cells was observed in the PGs and RCs. ZEB1 expression was significantly increased in periapical inflammatory tissues, and triple-positive cells for ZEB1, IL17, and CD4 were observed in the PGs and RCs. Notably, the number of ZEB1-IL17/CD4 triple-positive cells was significantly higher in PGs and RCs than in healthy controls; however, there was no significant difference between PGs and RCs (Fig. 2D). To further explore the important role of ZEB1 expression in Th17 cells in periapical lesions, we counted the proportion of ZEB1-positive cells among IL17^+^CD4^+^ Th17 cells (Fig. 2E). Notably, ZEB1-positive cells accounted for the majority of IL17^+^CD4^+^ Th17 cells, and there was no significant difference between the two periapical lesions groups.

### Colocalization of ZEB1 with p-STAT3 in human periapical lesions

**Fig. 3:**
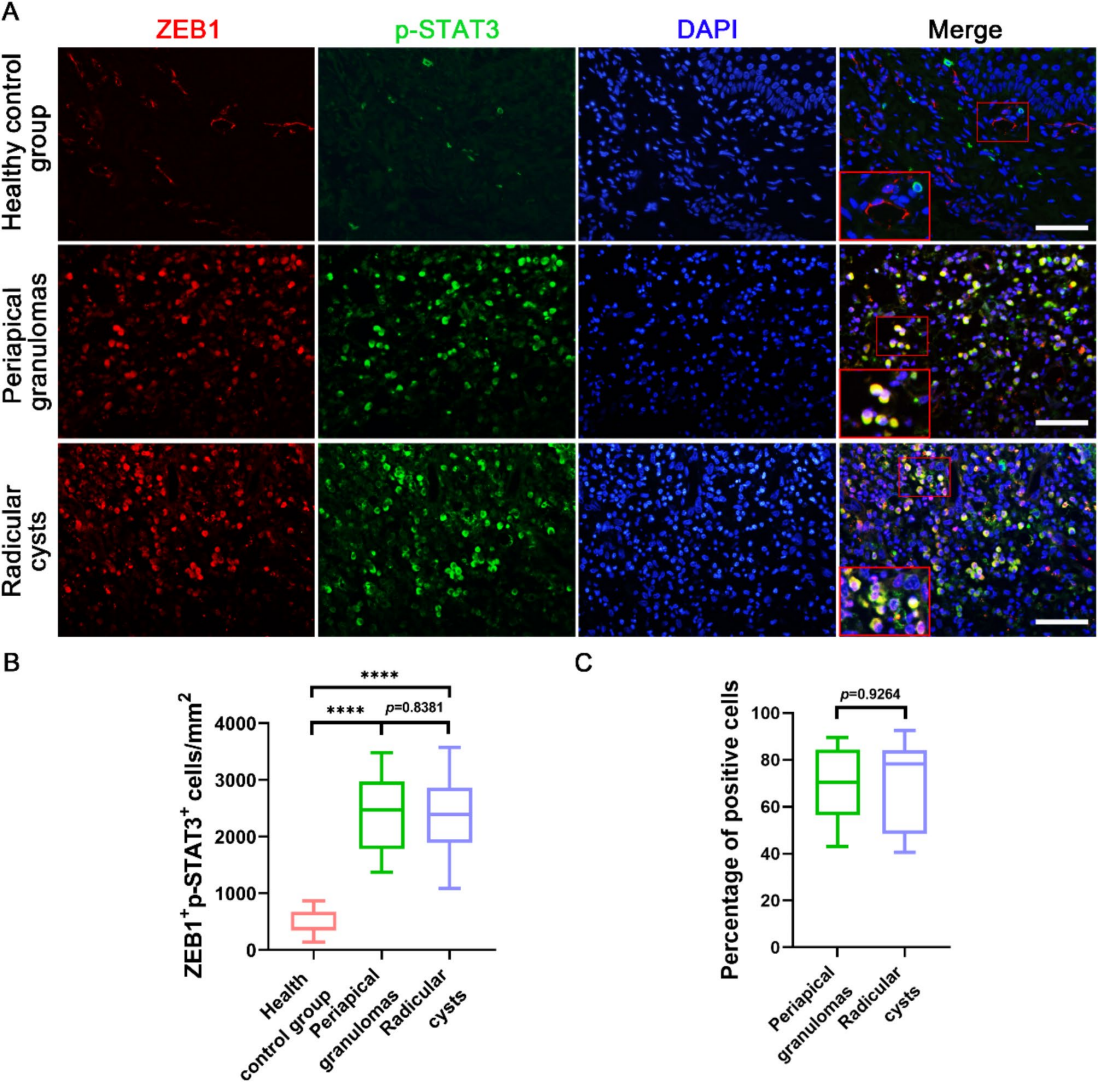
ZEB1 colocalized with p-STAT3 in human periapical tissues. **(A)** Immunofluorescence images identified the colocalization of ZEB1 and p-STAT3 in healthy control group, PGs and RCs. *Red*, ZEB1; *green*, p-STAT3; *blue*, DAPI. Scale bars = 50 μm. **(B)** Statistical analysis of ZEB1-p-STAT3 double-positive cells in every group via a box plot. Results are expressed as the number of positive cells per square millimeter. ****p <.0001. The *p*-value is statistically significant when *p* <.05. **(C)** Statistical analysis of ZEB1-positive cells among ZEB1 and p-STAT3 double-positive cells in every group via a box plot. Results are expressed as the percentage of positive cells. The *p*-value is statistically significant when *p* <.05

Immunoreactivity was also observed in healthy gingiva with a small number of p-STAT3-positive cells, but it was hardly co-expressed with ZEB1 in the same cells (Fig. 3A). There was large infiltration of ZEB1 and p-STAT3 double-positive cells in the PGs and RCs. Statistical analysis of ZEB1 and p-STAT3 double-positive cells revealed a significant increase in PGs and RCs compared with healthy controls, and there was no significant difference between the two inflammatory tissue groups (Fig. 3B). To verify the pervasiveness of the ZEB1-p-STAT3 axis in periapical lesions, we counted the proportion of ZEB1 and p-STAT3 double-positive cells accounting for ZEB1-positive cells (Fig. 3C). Complementing these findings, ZEB1 and p-STAT3 double-positive cells accounted for the majority of ZEB1-positive cells in the periapical lesions, and there was no significant difference between the PGs and RCs groups.

## Discussion

A recent meta-analysis of the global prevalence of periapical infection revealed that half of the world’s adults have at least one tooth with a periapical infection [27]. Persistent infiltration of the periapical tissue in the presence of bacteria and bacteriologically harmful substances evokes an immune response in the host, which in turn leads to persistent periapical lesions and eventual loss of the affected tooth [24]. Exploring the pathogenesis of periapical inflammation can help improve clinical treatment strategies for preserving the affected tooth.

Recently, as our understanding of EMT transcription factors has expanded, ZEB1 was found to play an important role in the transcriptional regulatory network that constitutes the differentiation, maintenance, and function of both myeloid and lymphoid lineage immune cells [12]. In this study, the expression of ZEB1 was significantly upregulated in PGs and RCs, but there were no significant differences between the two groups. However, in the larger periapical lesion tissue, which is the state of active periapical bone resorption, the expression of ZEB1 was elevated, which may be related to its role as a key regulator of energy metabolism in osteoclasts [13]. Nevertheless, there is a distinct difference in the types of cells expressing ZEB1 in the two periapical lesion types. While ZEB1 expression in PGs is primarily observed in inflammatory cells, in RCs, ZEB1 is also expressed in epithelial cells. ZEB1 expression is upregulated in epithelial tissues to promote cell migration or epithelial-to-mesenchymal transition during pathological processes such as cancer, chronic inflammation, or tissue repair [14, 28]. Notably, after the formation of PGs, persistent chronic inflammation, tissue necrosis, fluid accumulation, and epithelialization may contribute to the formation of RCs [24]. Given that molecular mechanisms driving epithelial cell proliferation are considered critical for cyst formation, we hypothesized that the upregulation of ZEB1 expression in epithelial cells of RCs may be related to its role in promoting the formation of the cyst wall.

The number of IL-17-positive cells is closely associated with the degree of bone destruction in periapical lesions [9]. Consistent with previous studies, the number of IL-17^+^ Th17 cells was significantly higher in PGs and RCs than in healthy gingival tissues [29]. Previous studies have shown that IL-17-positive cells are more abundant in PGs than in RCs, and that lesions with a sinus tract and a predominant multinucleated cell population exhibit a higher number of IL-17-positive cells [29]. The presence of a large number of IL-17-positive cells in PGs helps recruit other inflammatory cells, such as neutrophils and macrophages, which further secrete pro-inflammatory cytokines and matrix-degrading enzymes [7]. This sustained inflammatory process may promote tissue destruction and the formation of fluid-filled cavities, ultimately leading to the development of RCs [24]. In this study, IL-17^+^Th17 cells in the PGs and RCs groups showed a strong ZEB1-positive immune response. ZEB1 and IL-17 exhibit a close association and forming a feedback loop in tumor invasion, immune regulation, and inflammatory diseases [30–32]. In acute myeloid leukemia (AML) cells, ZEB1 directly promotes the development of Th17 cells, and conversely, the expansion of Th17 cells creates a pro-invasive phenotype, favoring the transcription of genes Interleukin-23 (IL-23) [30]. In asthma, during airway smooth muscle (ASM) remodeling, IL-17, through the ZEB1 pathway, reduces the expression of E-cadherin and increases the expression of vimentin, promoting the thickening of the ASM layer driven by Th17 cells in severe asthma [31]. Conversely, ZEB1 negatively regulates the transcription of the IL-2 gene, thereby partially suppressing Th1-mediated immune responses [32].

The STAT3 signaling pathway is important for the inflammatory response and osteoclast activity [18]. In two types of mouse periapical inflammation models, pulp cavity exposure and *Enterococcus faecalis* infection, p-STAT3 was positively correlated with the number of osteoclasts [19, 33]. The transcription factor ZEB1 and the STAT3 signaling pathway play crucial roles in EMT, tumor invasion, immune regulation, and chronic inflammatory responses [16, 20, 35]. In the tumor microenvironment, STAT3 and ZEB1 form a feedback loop that promotes EMT and enhances the invasive characteristics of tumor cells [21]. STAT3 can directly bind to the promoter region of the ZEB1 gene, promoting ZEB1 transcription, while ZEB1 regulates the production of cytokines such as IL-6 and TGF-β, which indirectly enhance STAT3 activation [21, 34]. In multiple sclerosis (MS), a prototypical organ-specific autoimmune disorder, downregulation of ZEB1 effectively suppresses STAT3 phosphorylation and subsequent IL-17 expression, thereby attenuating the differentiation of pathogenic Th17 cells [16]. In corneal inflammation, ZEB1 indirectly activates the STAT3 signaling pathway, thereby influencing late-stage wound healing [35]. In the present study, the number of ZEB1-p-STAT3 double-positive cells was significantly higher in periapical inflammatory tissues than in healthy tissues and did not differ in the two types of periapical lesions. Therefore, the ZEB1-STAT3 signaling axis plays an indispensable regulatory role in chronic apical periodontitis and exerts similar biological effects in the formation of both PGs and RCs.

In this study, we demonstrated for the first time that ZEB1 is highly expressed in IL-17^+^ Th17 cells and is significantly co-localized with p-STAT3 in periapical inflammatory tissues. However, this study has several limitations. The exact mechanism that how the ZEB1-STAT3 axis regulates IL-17^+^ Th17 cell activity and mediates periapical inflammation remains to be further explored, in order to clarify the functional differences of the ZEB1-STAT3 axis and IL-17^+^ Th17 cells in PGs and RCs.

## Conclusions

Our study investigating human apical periodontitis lesions, including PGs and RCs, identified ZEB1 as a potential participant associated with STAT3 activation and Th17 cells in the pathogenesis of apical periodontitis. Additionally, we observed that ZEB1 is expressed in the epithelial tissue of RCs, suggesting its potential involvement in the formation of the cystic wall. Our findings contribute to a better understanding of the potential mechanistic role of the ZEB1-STAT3 axis in human apical periodontitis and may offer new strategies for the targeted treatment of bone resorptive inflammatory diseases.

## Electronic supplementary material

Below is the link to the electronic supplementary material.

Supplementary Material 1

## Data Availability

The datasets used and/or analysed during the current study available from the corresponding author on reasonable request.
