# Supplementary material for: ZEB1 expression in Th17 cells correlated with p-STAT3 in human apical periodontitis
Source: BMC Oral Health. 2025 Feb 27;25:315. doi: 10.1186/s12903-025-05633-y (PMC11869427; doi:10.1186/s12903-025-05633-y)
Supplement: Supplementary file 1 — Supplementary Material 1 [file 12903_2025_5633_MOESM1_ESM.docx]

**Figures Legend**

**ZEB1 has been demonstrated to regulate immune cell function, and activation and phosphorylation of STAT3 is closely related to ZEB1 transcriptional activity.**

**Our previous study have comfirmed that Th17 cells are dynamically distributed in periapical disease tissues.**

**This study aimed to investigate the expression pattern of ZEB1 in Th17 cells and colocalization with p-STAT3 in the human apical periodontitis lesions.**

**Institutional review board approval to collect and reaserch human periapical lesions**

**(WHUSS, No.A15/2022)**

**Histology slides of human perapical lesions and healthy control tissues.**

**EXPERIMENTAL AND CONTROL GROUPS**

**Periapical granulomas (PGs) group, n=14;**

**Radicular cysts (RCs) group, n=12;**

**Healthy gingival tissues, n=13.**

**The expression of ZEB1 in Th17 cells in clinical human apical periodontitis lesions,**

**as well as the potential colocalization with p-STAT3.**

**HE staining, immunhistochemical staining and immunofluorescence labelling by laboratory staff.**

**1.Immunohistochemical analysis showed high expression of ZEB1;**

**2.Increased colocalization of ZEB1 with IL-17 and CD4 in infiltrated Th17 cells;**

**3.Increased colocalization of ZEB1 with p-STAT3.**

**This study reveals ZEB1 is a potential player correlated with STAT3 activation and Th17 cells in the pathogenesis of apical periodontitis.**

**The NSFC 82370914 and 81970919, the International Science and Technology Collaborative Project of Hubei Province 2024EHA062, the Fundamental Research Funds for the Central Universities 2042022kf1207 (L.Z.), the NSFC 82201042 and the Natural Science Foundation of Hubei Province 2022CFB658 (X.S.)**

**The authors deny any conflicts of interest related to this study.**

**Figure S1.** **Flowchart of designs and results of ZEB1 Expression in Th17 Cells Correlated with p-STAT3 in Human Apical Periodontitis.**

**
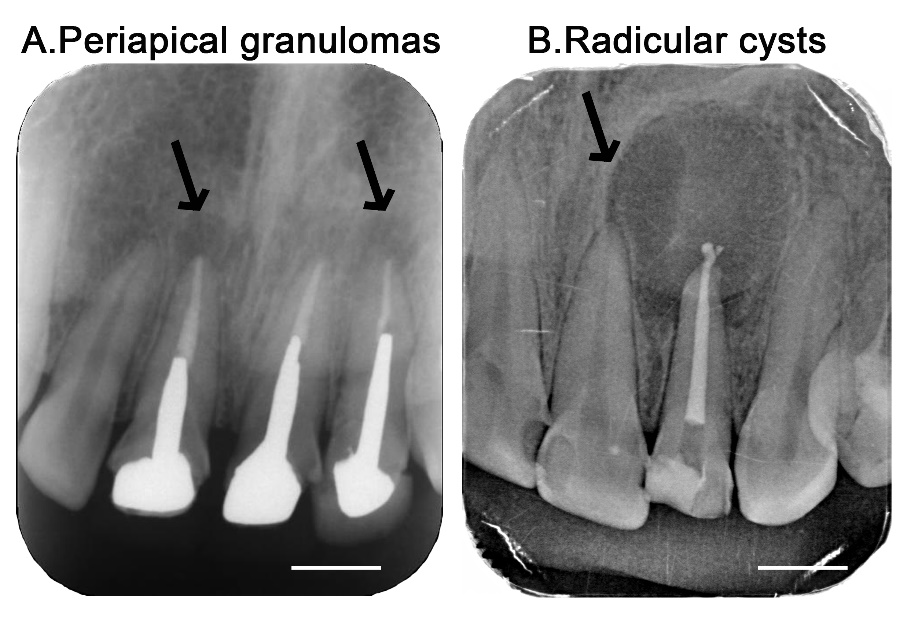
Figure S2.** **Representative X-ray radiographs of periapical lesions. (A)** Pericapical granulomas. **(B)** Radicular cysts. Scale bars = 5nm. The black arrow indicated the area of periapical lesions.
